# Thymidine Kinase 1 Upregulation Is an Early Event in Breast Tumor Formation

**DOI:** 10.1155/2012/575647

**Published:** 2012-06-19

**Authors:** Melissa M. Alegre, Richard A. Robison, Kim L. O'Neill

**Affiliations:** Department of Microbiology and Molecular Biology, Brigham Young University, Provo, UT 84602, USA

## Abstract

Prognostic markers play an important role in our understanding of tumors and how to treat them. Thymidine kinase 1 (TK1), a proliferation marker involved in DNA repair, has been shown to have independent prognostic potential. This prognostic potential includes the novel concept that upregulation of serum TK1 levels is an early event in cancer development. This same effect may also be seen in tumor tissue. In order to demonstrate that TK1 upregulation is an early event in tumor tissue formation, tissue arrays were obtained and stained for TK1 by immunohistochemistry. Using a progressive breast tissue array, precancerous tissue including breast adenosis, simple hyperplasia, and atypical hyperplasia stained positive for TK1 expression. Different stages of breast carcinoma tissue also stained positive for TK1 including nonspecific infiltrating duct, infiltrating lobular, and infiltrating duct with lymph node metastasis carcinomas. This indicates that TK1 upregulation is an early event in breast carcinoma development, and may be useful in identifying precancerous tissue. Further work is needed to better understand the differences seen between TK1 positive and negative tissues.

## 1. Introduction

Studies have shown that the early detection of breast cancer leads to better patient prognosis and a greater five-year survival rate. Diagnostic and prognostic markers play a key role in classifying tumors and determining the best treatment plan for a patient. The most widely used and established prognostic markers for breast cancer recurrence are tumor size, tumor grade, lymph node involvement, and tumor hormone receptor status. These indicators, although well established, are all related to tumor aggressiveness. Recent evidence has shown that proliferation markers, such as Ki-67 and proliferating cell nuclear antigen (PCNA), may have independent prognostic value [1–3]. Although these proliferation markers have potential, recent studies indicate that thymidine kinase 1 (TK1), another marker associated with proliferation, may be a better prognostic marker than either Ki-67 or PCNA [4, 5]. 

Thymidine kinase 1 (TK1) has been studied extensively, primarily as a diagnostic biomarker for a variety of cancer types. TK1 is a nucleotide salvage pathway repair enzyme that is primarily responsible for the phosphorylation of thymidine to thymidine monophosphate. TK1 is associated with proliferating cells and is primarily elevated during S phase [6, 7]. As a biomarker, higher serum TK1 activity levels correlate with a more advanced cancer stage and grade [8–10]. Serum TK1 levels also show prognostic potential as their levels help predict future relapse at the time of primary diagnosis in breast and colorectal cancer patients [11, 12]. 

Similar trends have been found between tumor tissue and TK1 expression levels. One study demonstrated that breast cancer patients who later showed recurrence initially had higher primary tumor TK1 levels when compared to those patients who did not show recurrence [13]. Furthermore, breast cancer patients with either high or intermediate TK1 activity in their tumors showed rapid disease progression and poorer prognosis as compared to patients with low TK1 activity in their tumors [14]. Tumor TK1 levels, similar to serum TK1 levels, also correlate with both stage and grade [15]. Tumor TK1 has also been compared with both Ki-67 and PCNA. Although there is a significant correlation between PCNA and TK1 staining of breast cancer tissue, TK1 showed a significant correlation with stage and grade while PCNA did not, indicating that TK1 might be a more accurate marker for diagnosis and prognosis [4]. Similarly, there is a significant correlation between Ki-67 and TK1 in breast cancer tissue when compared to normal tissue; however, due to early upregulation of TK1 as compared with Ki-67, TK1 may be a more accurate prognostic marker [16, 17]. 

TK1 upregulation as an early event of cancer is a novel concept that has been addressed by only a few recent studies. One such study involving a health screening of 8,135 people found that 89.2% of persons with elevated serum TK1 levels had diseases linked to risk for pre-/early cancerous progression, including one individual who developed liver carcinoma 13 months after the health screening [18]. Similar studies have also shown that recurrence can be detected by elevated serum TK1 levels as early as 1–6 months before the clinical onset of relapse [19]. These studies show the early nature of serum TK1 levels in tumor development. This study seeks to determine whether, similarly to serum TK1, tumor TK1 upregulation is an early event in tumor development and may aid in the identification of precancerous tissue.

## 2. Materials and Methods

### 2.1. Patients and Specimens

 Tissue arrays containing tissue from normal (*n*  =  56), adenosis (*n*  =  22), and breast carcinoma (*n*  =  97) patients as well as a progressive breast array (Cybrdi Inc., Frederick MD) were analyzed for TK1 expression. Breast carcinoma tissue included simple carcinoma (*n*  =  30), infiltrating duct carcinoma (*n*  =  41), medullary carcinoma (*n*  =  12), scirrhous carcinoma (*n*  =  11), and infiltrating lobular carcinoma (*n*  =  3).

### 2.2. Immunohistochemistry

 Tissue arrays were stained using an anti-TK1 mouse monoclonal antibody (CB001), which we previously demonstrated to be highly specific to TK1 [20]. Using this antibody, histological slides were stained using the following procedure. Briefly, formalin-fixed paraffin-embedded specimens were prepared by deparaffinization and rehydration. To retrieve antigenicities of TK1, specimens were boiled in 0.01 M sodium citrate buffer (pH 6.0) for 12 minutes and allowed to cool at room temperature for 20 minutes. The endogenous peroxidase activity was blocked by immersion in 3% H_2_O_2_ in methyl alcohol at room temperature for 20 minutes. The slides were then washed in phosphate-buffered saline (PBS; pH 7.2) and blocked in 10% normal horse serum for 30 minutes. After blocking, the slides were incubated at room temperature for 3 hours with either anti-TK1 mouse monoclonal antibody (diluted 1 : 100) or isotype control (0.6 *μ*g/*μ*L, mouse IgG, Upstate Company, 12-371). Slides were washed with PBS and then incubated with a biotin-conjugated anti-mouse secondary antibody (ABC kit, Vector Lab Inc.) at room temperature for 30 minutes. After PBS washing, slides were incubated for 30 minutes, at room temperature, with Streptavidin-Peroxidase (ABC Kit, Vector Lab Inc.) and then washed again in PBS. Diaminobenzidine (Vector Lab Inc.) was used as a chromagen, and the slides were counterstained with haematoxylin.

### 2.3. Statistical Analysis and Scoring

 Specimens were scored by three pathologists, and a consensus score of positive, weak positive, or negative was compiled. A positive score indicated cytoplasmic staining of TK1 in 5–25% of tumor cells. If some signal was detected but was insignificant when compared to the isotype control, it was given a weak positive score. A negative score indicated no staining. All blood vessels and fibrous tissue cores were excluded from statistical analysis. A chi-square test of independence was applied to compare the scores of normal and malignant tissues. Due to the limited number of cases in the progressive breast array, no statistical analysis on this array could be performed. Differences with *P* < 0.05 (two-sided) were regarded as statistically significant.

## 3. Results and Discussion

TK1 expression was found to be significantly different (*P* < 0.001) between normal breast and breast carcinoma tissue. A total of 73 breast carcinoma tissues (79%) were positive for TK1 expression while only 18 normal breast tissues (36%) scored positive. Breast tissue was also stained using an isotype control (mouse IgG, Upstate Company), and all breast tissue was found to be negative. Since these normal tissues were retrieved from the margins around a tumor and were considered pathologically normal, we sought to determine if tissue from noncancerous individuals yielded similar results. Interestingly, there was no TK1 staining in any breast tissue obtained from noncancerous individuals, called FDA-approved true normal tissue (data not shown). Therefore, these 18 TK1 positive normal tumor margins may not be false-positive results, but rather precarcinoma tissue, which is considered pathologically normal tissue by current standards. Further work is needed to better understand these potential differences.

**Figure 1:**
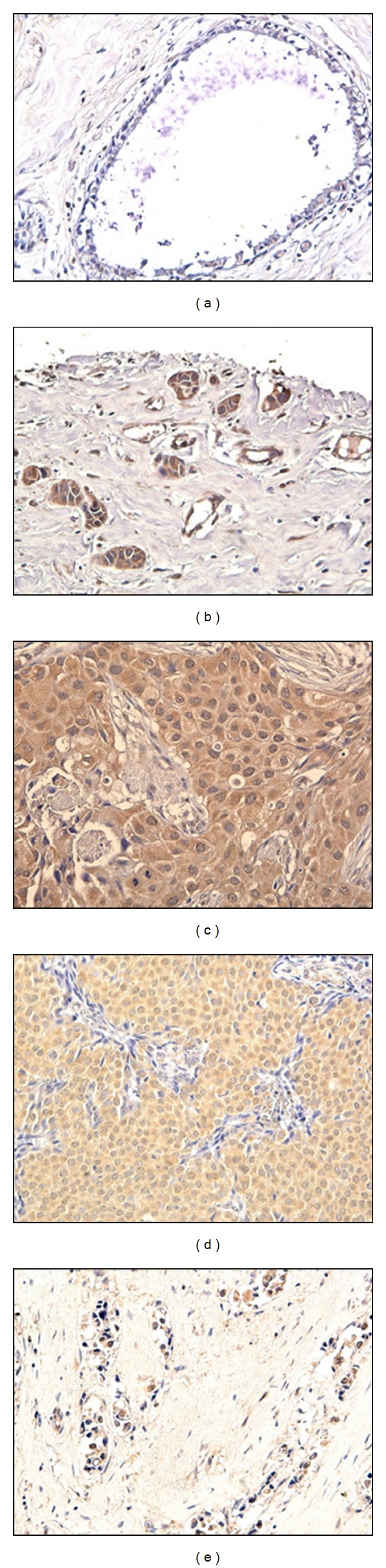
TK1 breast tissue staining. (a) No TK1 staining was found in most normal duct tissue. TK1 positive staining could be found in the cytoplasm of tumor cells of (b) simple carcinoma, (c) infiltrating duct carcinoma, (d) medullary carcinoma, and (e) sclerosing carcinoma tissues.

TK1 expression was also found to be significantly different (*P* = 0.013) between the different types of breast carcinoma tissue. A chi-square test of independence was applied to compare the scores of the various types of breast carcinoma tissue. The Pearson chi-square value was 22.452, using 10 degrees of freedom, and the two-sided *P*-value was 0.013. The results are summarized in Table 1 and typical staining can been seen in Figure 1. In summary, infiltrating lobular carcinoma and scirrhous carcinoma tissues all stained positive for TK1, while 66–83% of simple, infiltrating duct, and medullary carcinoma tissues stained positive for TK1. Further studies with larger sample sizes may further elucidate the differences between these tissue types. 

**Figure 2:**
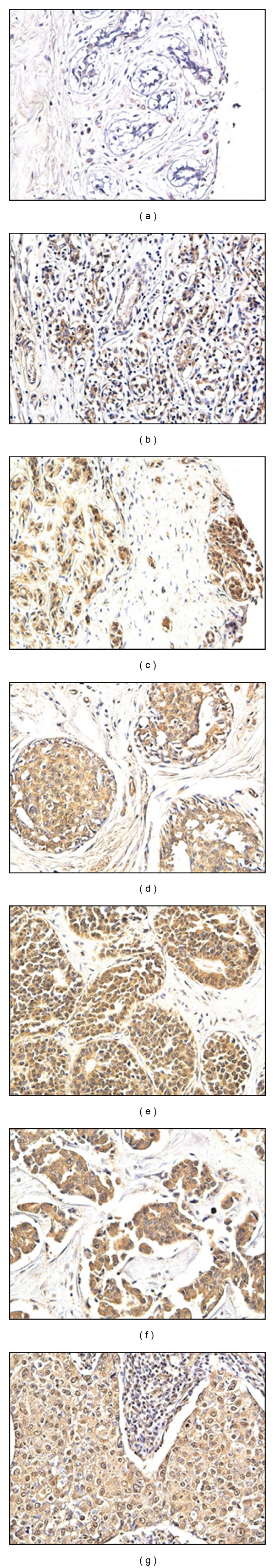
Breast progressive array staining. (a) No TK1 staining was found in normal lobule breast tissue. However, TK1 staining was found in proliferating duct epithelial cells of precancerous tissue including, (b) breast adenosis, (c) breast adenosis with mild atypical hyperplasia of duct epithelium, and (d) moderate atypical hyperplasia of duct epithelium. Positive TK1 staining in the cytoplasm of tumor cells was also found in cancerous tissue such as, (e) intraductal carcinoma, (f) infiltrating duct carcinoma, and (g) medullary carcinoma tissues.

In addition to the TK1 positive breast carcinoma tissues, 4 breast adenosis tissues (22%) were also found to be positive for TK1 expression. These 4 positive precancerous tissues were the first indication that TK1 expression may be an early event in tumor development. To pursue this hypothesis, we obtained a progressive breast array. This progressive breast array included tissue from different tumor developmental stages, such as normal, adenosis, and atypical hyperplasia, moderate atypical hyperplasia, severe hyperplasia, nonspecific infiltrating duct carcinoma, infiltrating lobular carcinoma, and infiltrating duct carcinoma with lymph node metastasis. The results are summarized in Table 2 and typical staining can be seen in Figure 2. The proliferating epithelial cells of some cases of breast adenosis were positive for TK1 expression as well as breast tissue with simple or atypical hyperplasia. As previously seen, most breast carcinoma tissues were also positive for TK1 expression while no breast tissue stained positive with an isotype control. It appears from this progressive array that since TK1 is found in precancerous tissue, TK1 upregulation is an early event in breast tumor development. These results support the previous conclusion that in some cases, there may be a difference between true normal tissue from noncancerous patients and the pathologically normal tumor margins. Further studies are needed to elucidate the differences between both the normal tumor margins and precancerous tissues that were positive for TK1 and those that were negative. Perhaps, the prognostic value of TK1 may be of help in identifying those precancerous tissues which are of greatest risk to the patient. Therefore, TK1 expression is an early event in tumor development and may aid in the identification of precancerous tissue.

## 4. Conclusion

The aim of this study was to determine if TK1 upregulation is an early event in tumor development. From the progressive breast array, it can be seen that in many cases of breast cancer, TK1 is upregulated in precancerous tissue and remains elevated in correlation to cancer stage. This confirms earlier research that indicated that elevated TK1 levels correlated with early recurrence. Although not elevated in all tumors, TK1 appears to be upregulated as an early event in most tumors and therefore can possibly be used in connection with other diagnostic and prognostic techniques to improve patient outcome. These results also indicate that the TK1 positive pathologically normal tumor margins may in fact be tumor cells that have escaped pathological identification. This preliminary research may indicate that TK1 can be used to identify possible malignant cells, which have evaded pathological detection during surgical removal. Unfortunately due to the anonymity of these patient samples, we have been unable to determine if TK1 positive tumor margins are of clinical significance. Further research would be required to establish if these TK1 positive cells are in fact a result of the tumor tissue. Overall, it appears that TK1 has diagnostic and prognostic potential in identifying breast tumor tissue as well as precancerous tissues. The ability to identify tumor tissue during the early stages of development is of significant value. Therefore, the histological identification of tumors utilizing TK1 suggests promising prognostic and diagnostic potential in breast cancer tissue.

## Figures and Tables

**Table 1:** TK1 scoring of different types of carcinoma tissue.

Diagnosis	Negative	Weak positive	Positive	Total
Simple carcinoma	3	2	25	30
Infiltrating duct carcinoma	11	4	26	41
Medullary carcinoma	4	0	8	12
Scirrhous carcinoma	0	0	11	11
Infiltrating lobular carcinoma	0	0	3	3

Total	18	6	73	97

**Table 2:** TK1 scoring of breast progressive array.

Pathological types of breast tissues	Negative/positive
Normal tissues	−
Breast adenosis	−
Sclerosing adenosis	−/+
Atypical hyperplasia	−/+
Infiltrating lobular carcinoma	+
Infiltrating carcinoma	+
Infiltrating carcinoma with lymph node metastasis	+
